# A longitudinal mixed methods evaluation of a facilitation training intervention to build implementation capacity

**DOI:** 10.3389/frhs.2024.1408801

**Published:** 2024-09-13

**Authors:** Veronica-Aurelia Costea, Annika Bäck, Anna Bergström, Andreas Lundin, Henna Hasson, Leif Eriksson

**Affiliations:** ^1^ PROCOME Research Group, Department of Learning, Informatics, Management and Ethics, Karolinska Institutet, Stockholm, Sweden; ^2^ Unit for Implementation and Evaluation, Center for Epidemiology and Community Medicine, Stockholm, Sweden; ^3^ Unit for Health Care Analysis, Center for Epidemiology and Community Medicine, Stockholm, Sweden

**Keywords:** facilitation, implementation, capacity-building, knowledge translation, self-efficacy, normalization, intervention research, longitudinal study

## Abstract

**Background:**

There is a demand for facilitators who can ease the collaboration within a team or an organization in the implementation of evidence-based interventions (EBIs) and who are positioned to build the implementation capacity in an organization. This study aimed to evaluate the results the Building implementation capacity for facilitation (BIC-F) intervention had on the participants' perceived knowledge, skills, and self-efficacy to facilitate implementation and the normalization of a systematic implementation model into their work routines, and its use into their respective organizations.

**Methods:**

The BIC-F intervention was delivered to 37 facilitators in six workshops, which focused on teaching participants to apply a systematic implementation model and various facilitation tools and strategies. A longitudinal mixed methods design was used to evaluate the intervention. Data was collected pre- and post-intervention using questionnaires and semi-structured interviews grounded on the Normalization Process Theory (NPT). Quantitative data were analyzed using descriptive (mean, SD) and inferential (paired t-tests) methods. Qualitative data were analyzed using deductive content analysis according to NPT.

**Results:**

An increase in the participants' perceived knowledge, skills, and self-efficacy was observed post-intervention. Normalization of the systematic implementation model in the participants' work routines was in an early phase, facilitated by high coherence, however, other NPT mechanisms were not sufficiently activated yet to contribute to full normalization. In the organizations where participants initiated the normalization process, they were still working towards achieving coherence and cognitive participation among relevant stakeholders.

**Conclusion:**

The intervention had positive results on the participants' perceived knowledge, skills, and self-efficacy and these recognized the value of a systematic implementation model for their practice. However, further efforts are needed to apply it consistently as a part of their work routines and in the organization. Future interventions should provide long-term support for facilitators, and include methods to transfer training between organizational levels and to overcome contextual barriers.

## 1 Introduction

Various theories, models, and frameworks have described the factors, processes, and strategies influencing the successful implementation of evidence-based interventions (EBIs) (1). Facilitation is an overarching strategy suggested to support the implementation of EBIs and constitutes the “active ingredient” in the implementation process in the integrated-Promoting Action on Implementation in Health Services (i-PARIHS) framework (2). The i-PARIHS framework proposes that facilitators play a key role in the implementation of EBIs by supporting the tailoring of implementation strategies to the characteristics of the innovation (e.g., clarity, relative advantage, and usability), its recipients (e.g., their knowledge and skills, motivation, and resources) and the inner and outer context (e.g., leadership support, culture, previous experiences with implementation, and policy) in which implementation takes place (2). A *facilitator* is thus a person who enables the change by easing collaboration and participatory problem-solving between stakeholders. The facilitator is uniquely positioned to help leaders and staff customize the implementation plan by choosing strategies appropriate for the organizational context and stakeholder dynamic (3–6). Facilitators can thus intentionally set cognitive and social mechanisms in motion to promote the normalization of EBIs (7). Teams and organizations where facilitation techniques are employed are more likely to make complex practice changes by, for example, implementing more EBIs (e.g., guidelines) (8), increasing adherence to them (9), or by improving the delivery of care (10).

Aside from supporting the normalization of new behaviors, facilitation has been highlighted to build internal implementation capacity in organizations (11), both through the specific roles and activities that facilitators carry out (12), and through their position, which enables them to train organization staff in implementation (6). For implementation to happen, *normalization*, i.e., the embedding of interventions in practice, is required. The Normalization Process Theory proposes four mechanisms that need to be triggered to achieve normalization (13). *Coherence* involves the work individuals and groups do when faced with the challenge of operationalizing a set of practices. *Cognitive participation* entails the relational work people do to build and sustain a community of practice around a new technology or complex intervention. *Collective action* encompasses the practical implementation of a set of practices. *Reflexive monitoring* is the appraisal work people do to assess and understand how a new set of practices impacts them and others. Each mechanism comprises four subconstructs (called submechanisms in this article).

The effectiveness of facilitation hinges on facilitators' knowledge, skills, and self-efficacy. Reviews of the healthcare services and implementation science literature have listed and described the type of knowledge, skills, and characteristics that facilitators should possess (14, 15). Apart from hard skills (e.g., searching, retrieving, appraising, and synthesizing evidence), facilitators should also demonstrate a wide range of soft and open skills, commonly falling within the categories of self-awareness, self-management, social and cultural awareness, and relationship management (14, 16).

How to best prepare facilitators for their role is not fully understood (17). Facilitation training can increase the chance of successful implementation and normalization of EBIs, which may lead to positive outcomes for patients in the long run (18). In contrast, training that does not match the facilitators' needs and expectations (19) or is insufficient can create barriers in the implementation process (19, 20). Although there are some interventions aiming to train professionals as facilitators (21–23), very few longitudinal evaluations of such interventions are described in the literature.

This longitudinal study aimed to evaluate the results the *Building implementation capacity for facilitation* (BIC-F) intervention had on the participants' knowledge, skills, and self-efficacy to facilitate implementation and the normalization of a systematic implementation model into their work routines, as well as its use into their respective organizations.

## 2 Methods

This longitudinal study used a mixed methods sequential explanatory design, in which the quantitative results are explained using the qualitative findings (24). The collected quantitative data was analyzed and used as a starting point to develop the interview guide. The integration of the quantitative and qualitative results is carried out in the discussion section of the manuscript. The Template for Intervention Description and Replication (TIDieR) (25) was followed, and the filled-out checklist is provided as a supplementary file (Supplementary Material: TIDieR Reporting Standards Checklist).

### 2.1 Setting

The study was conducted in Region Stockholm, Sweden. The development, implementation and evaluation of the intervention were carried out by members of the Unit for Implementation and Evaluation, at the Center for Epidemiology and Community Medicine (CES), responsible for providing implementation and evaluation support to healthcare organizations in the region.

### 2.2 Intervention development and delivery

#### 2.2.1 Development of learning objectives

Learning objectives were developed based on two sources of knowledge: scientific and practical. Firstly, a literature review was carried out to identify the most common knowledge and skills in scientific literature needed for effective facilitation. Secondly, to gather input on the learning objectives from practice, the method of *adaptive reflection* (21, 26) was applied in two workshops held in the autumn of 2018. One workshop was held with the whole Unit for Implementation and Evaluation (*n* = 15) and another with professionals having a facilitator role in organizations in Region Stockholm (*n* = 13). The participants in the workshops were asked to answer the following questions: (1) *Which knowledge, skills, attitudes, and behaviors do facilitators need to make the implementation of new methods easier for others?*; (2) *Which contextual conditions should be created so that facilitators, using the knowledge, skills, attitudes and behaviors identified in the first step, can make the implementation of new methods easier for others?*

The findings from the literature review and the workshops were categorized, and three learning objectives were formulated, employing Bloom's taxonomy (22). After the intervention, the participants were expected to be able to:

• Apply an evidence-based model for behavior change as a tool for implementation;
• Support implementation work by motivating, communicating, and giving positive feedback;
• Support the sustainability of implementation by follow-up, adaptation, and alignment.

#### 2.2.2 Intervention content and implementation

The BIC-F intervention was developed at CES by a team of five experts with extensive experience developing and delivering implementation and facilitation trainings.

The content was developed to achieve the intervention's learning objectives, and its delivery was structured according to the pedagogical theories of Kolb, Biggs, and Bloom (23, 27, 28). The BIC-F intervention had four core components. **Core component one** entailed lectures and exercises to increase the participants' knowledge and skills in systematic implementation. The focus was that participants learn to apply a systematic implementation model to support behavior change (29). The model includes six steps: (i) describe the implementation goal; (ii) specify target behavior(s); (iii) for each behavior, analyze what is needed for behavior change to occur; (iv) choose implementation strategies; (v) apply implementation strategies; and (vi) monitor occurrence of the target behavior (29). **Core component two** consisted of lectures and exercises to increase knowledge and skills in facilitation. **Core component three** consisted of practical work on an implementation plan in-between workshops and in collaboration with organizational stakeholders by anchoring it with them. **Core component four** entailed peer support and feedback on performance from workshop leaders and other training participants. The participants interacted and collaborated with each other and with course leaders throughout the workshops. Workshop leaders also carried out group supervision during the workshops and individual supervision in-between workshops. The activities and content of the intervention are described in Table 1.

**Table 1:** Activities and content of the building implementation capacity for facilitation (BIC-F) intervention.

Session	Activity and content
Workshop 1	- Lecture: Introduction to implementation, a systematic implementation model and facilitation - Exercise: Brainstorming on facilitation skills and attributes (as one group)
In-between workshops	- Initiate an implementation plan for own case
Workshop 2	- Lecture: Applying a systematic implementation model in an organization. - Exercise: Applying the systematic implementation model to fictitious case - Group supervision (3–4 participants): reflection on participants’ application of the systematic implementation model on own case
In between workshops	- Continue developing an implementation plan for case and reflect on own facilitation skills and attributes
Workshop 3	- Lectures: Providing feedback; Applying the systematic implementation model: elaboration on the first three steps of the model; Brainstorming techniques - Group supervision (3–4 participants): reflection on participants’ application of the systematic implementation model on own case
In between workshops	- Anchoring the implementation plan with key stakeholders and revise implementation plan according to feedback from stakeholders - Individual supervision: discussion of implementation plan and other topics relevant to the facilitator (30 min)
Workshop 4	- Lecture: Communication; Applying the systematic implementation model: elaboration on the last three steps of the model - Exercise: training on how to communicate about the implementation case using the elevator pitch technique - Group supervision (3–4 participants): reflection on participants’ application of the systematic implementation model on own case
In between workshops	- Anchoring the implementation plan with key stakeholders and revise plan according to feedback from stakeholders - Individual supervision: discussion of implementation plan and other topics relevant to the facilitator (30 min)
Workshop 5	- Lecture: Change resistance - Exercise: Applying facilitation tools relevant for change resistance and role-play on change resistance
In between workshops	- Home assignment: Using the introduced facilitation tool and practice on facing change resistance in conversation
Workshop 6	- Lecture: sustaining implementation through follow-up, adaptation, and alignment - Exercise: applying the systematic implementation model to fictitious case (3–4 participants) - Reflections on lessons learned (as one group)

Initially, the content was designed for four in-person workshops (two full days and two half-days). However, because of the COVID-19 pandemic, the intervention was delivered digitally in six half-day workshops. Three cohorts participated and the first was enrolled in autumn 2020 (*n* = 10), the second in spring 2021 (*n* = 12), and the third in autumn 2021 (*n* = 15). Digital tools such as Mentimeter (30) and Padlet (31) were used as examples of tools useful for facilitation.

After each workshop, participants received PDFs of lecture slides and worksheets. Between workshops and after the workshop series participants were invited to reach out if they needed more support from the workshop leaders. Three months after the intervention, one of the workshop leaders had telephone conversations with the participants to allow them to reflect on their situation and ask questions.

### 2.3 Intervention participants

An announcement of the training and the requirements for participation was posted on a webpage dedicated to healthcare and public health in Region Stockholm (www.folkhalsoguiden.se). Interested individuals could sign up using the enrolment form on the website. One of the workshop leaders subsequently contacted and interviewed them. Participants were admitted into the training program if (i) they had a facilitating function in the public organization where they worked; (ii) they had an ongoing or upcoming implementation project they could work on during the training; (iii) their line manager supported their participation; and (iv) they could participate in all the workshops. Three cohorts (with 37 participants in total) were enrolled in the study.

### 2.4 Data collection

The data were collected between autumn 2020 and autumn 2022. The quantitative data collection for the three cohorts consisted of questionnaires administered pre-intervention, post-intervention, and as a follow-up six months after the information Qualitative data was collected through semi-structured individual interviews carried out approximately one year after the intervention.

#### 2.4.1 Quantitative data

Quantitative data were collected using the online survey tool KI Survey®. The participants were followed longitudinally, with each receiving a unique identification number.

Knowledge to facilitate implementation (pre- and post-intervention questionnaires) was evaluated with a five-item composite measure. Items reflected the intervention learning objectives and were based on a previously established instrument (32). The participants were asked to rate their perceived knowledge to support the planning, implementation, adaptation, follow-up, and coordination of EBIs. Example-statements were: “I have enough knowledge to facilitate the planning of the implementation of new work routines” and “I have enough knowledge to facilitate the follow-up of the implementation of new work routines”. The response format of the items was a visual analog scale (VAS) from *0 - Completely disagree* to *100 - Completely agree*.

Skills in facilitation (pre- and post-intervention questionnaires) were evaluated with a five-items composite measure also based on a previous instrument (32). The participants were asked to rate their perceived skills to motivate stakeholders, communicate the implementation, express an understanding of the problems the stakeholders experience during implementation, manage conflicts, and provide constructive feedback. Example-statements were: “I have the required skills to motivate the group to implement new work routines” and “I have enough skills to manage conflicts”. The items were measured using a VAS response format from *0 – Completely disagree* to *100 – Completely agree*.

Self-efficacy to facilitate implementation (pre-, post-intervention and six months follow-up questionnaires) refers to individuals' belief in their ability to perform a specific task (33) was measured using an adapted version of the general self-efficacy scale (34). The original scale consists of ten items with a four-point Likert response format. We used the ten-item structure, but the response format was replaced with a VAS from *0-Completely disagree* to *100-Completely agree* to comply with Bandura's recommendations regarding response formats (33). The scale's face validity was checked by experts from the Unit for Implementation and Evaluation at CES, which led to changing the wording of some items. Example-items: “At present, I am confident that, in my role as facilitator, I can always manage to solve difficult problems if I try hard enough” and “At present, I am confident that, in my role as facilitator, I can usually find several solutions when I am confronted with a problem”.

Normalization of the systematic implementation model (six months follow-up questionnaire) was measured using three general items and the S-NoMAD scale. The three general items were used to inquire about the participants' familiarity with the systematic implementation model, whether the use of the systematic implementation model feels like a natural part of their work routine, and if the systematic implementation model can become a natural part of their work routine (35). The items were measured using a VAS response format from 0 to 10 (35). The S-NoMAD scale contains a total of 20 items covering the submechanisms of NPT, explained separately (Supplementary Material: Data collection instruments, pp. 13–14). The response format of the scale ranges from 1-*Strongly agree* to 5-*Strongly contradict*. Example items are: “I can see how the intervention differs from the usual ways of working” and “There are key people who drive the intervention forward and get others involved”. The scale has shown good reliability in the original validation study (Cronbach's alpha of. 79) (35). Moreover, three response alternatives covering situations not relevant to the respondent were provided for each item (*6 - Not relevant for this role*, *7 - Not relevant at the moment*, *8 - Not relevant for the intervention*) (35).

The pre-intervention questionnaire also contained items on individual-level factors (learning motivation, training motivation) and contextual-level factors (the participants' perception of the implementation climate and collegial support), to understand transfer of training (36). Learning motivation has been defined as a desire of the participant to learn the training content (37) and was measured with a single item “I am motivated to learn what will be presented in the course”. Training motivation is the intensity of a participant's commitment to perform in training situations (37) and was measured single item “I am prepared to make great efforts to develop myself in the role of facilitator during the course”. Implementation climate refers to the participants' individual “perceptions of the extent to which their use of a specific innovation is rewarded, supported, and expected within their organization” (38, 39). Implementation climate was measured pre-intervention with an instrument consisting of five items inspired by a previously validated scale (38). Collegial support refers to the perceived support participants think they will receive from the group they facilitate (40). Collegial support was measured pre-intervention using a single statement, “I think that the group I facilitate will support me to apply the competencies I will learn during the training”. All items were measured using a VAS response format from *0 (Completely disagree)* to *100 (Completely agree)*.

#### 2.4.2 Qualitative data

Semi-structured interviews were conducted approximately one year after participants had completed participation in the intervention. The questions in the interview guide focused on the normalization of the systematic implementation model in the participants' routines, and in the organization. Therefore, specific questions were formulated to elicit responses regarding the normalization mechanisms identified by Normalization Process Theory (41).

A convenience sampling method was used to recruit interview participants. All intervention participants in the three cohorts were invited to participate in the interviews. They received an email seven months after the last workshop. One follow-up email was sent to those who did not reply to the first email. If they accepted the invitation, they were sent the informed consent form. Before the interviews, participants' verbal consent to participate in the interviews was recorded, and the recording was stored separately from the data. The interviews were carried out via Microsoft Teams and were audio-recorded.

### 2.5 Data analysis

#### 2.5.1 Quantitative data

Quantitative data from the three cohorts was aggregated and analyzed descriptively in SAS (v. 9.4). Cronbach's alpha at T1 and T2 were calculated for the knowledge and skills composite measures. The instrument measuring knowledge to facilitate implementation had a Cronbach's alpha of.95 at T1 and of.91 at T2. The instrument measuring skills in facilitation had a Cronbach's alpha of.91 at T1 and.81 at T2. Paired-sample t-tests were carried out to analyze whether knowledge and skills changed between pre-intervention and post-intervention.

Sum scores, which are recommended to analyze the general self-efficacy scale (34), and Cronbach's alpha (.94) were calculated for the self-efficacy scale. Prior to analyzing the S-NoMAD scale, the scores for the item *Relational Integration 1* were reversed because of its negative wording. Mean scores and standard deviation (SD) were computed for the three general questions, the S-NoMAD scale, and for each S-NoMAD subscale of the questionnaire sent six months post-intervention.

#### 2.5.2 Qualitative data

The interviews were transcribed verbatim by a professional transcriber. Qualitative data analyses were conducted in QSR NVivo v11 (42) using deductive qualitative content analysis (43). Initially, to achieve a common understanding of the NPT submechanisms in relation to the data, three of the authors coded three interviews using NPT constructs. The first author coded the rest of the interviews. The data under each code were discussed with the second author to check if the meaning units reflected the meaning of the code, thus increasing coding reliability. The meaning units for each code were then summarized. Meaning units reflecting inner and outer context and which did not refer directly to the use of the systematic implementation model were analyzed using a general inductive approach. This approach involved creating codes based on the data, which were then grouped into broader categories, summarized, and presented as an introduction to Contextual integration, as many of the topics discussed about the model were also brought up to describe the general implementation climate.

### 2.6 Ethical considerations

This study was reviewed by the Swedish Ethical Review Authority, who considered that it did not need any ethical approval (Ref no: 2020-03601). All participants were treated in accordance with the ethical guidelines. For the qualitative data collection, the participants were informed about the purpose of the study and that they could withdraw from the study at any time, without explanation. Participation in the study was voluntary and no monetary compensation was offered.

## 3 Results

### 3.1 Participants

The total sample of 37 intervention participants comprised of individuals with positions titled *development leader*, *method developer*, *quality developer*, *quality leader* and similar, who worked in health care (e.g., at hospitals and health care administration) and for local and national public organizations (e.g., Social Services Administration, the Swedish Civil Contingency Agency, and the Swedish Police). The participation rates in the surveys and interviews for each cohort are presented in Table 2. All participants (*n* = 37) completed the pre-intervention questionnaire. The post-intervention questionnaire was answered by 86.5% (*n* = 32) of the participants and 45.9% (*n* = 17) answered the six months follow-up questionnaire. Seventeen participants took part in the interviews.

**Table 2:** Description of the three cohorts and questionnaire response rates.

Cohort participation	Cohort			Total
	1	2	3	
Intervention delivery	October 2020–February 2021	March 2021–May 2021	September 2021–December 2021	
Number of participants	10 Healthcare: 4 Other public organizations: 6	12 Healthcare: 8 Other public organizations: 4	15 Healthcare: 12 Other public organizations: 3	37
Questionnaire response rates	*n* (%)	*n* (%)	*n* (%)	*n* (%)
Pre-intervention	10 (100)	12 (100)	15 (100)	37 (100)
Post-intervention	10 (100)	12 (100)	10 (66.6)	32 (86.5)
Six months follow-up	7 (70)	7 (58.3)	3 (27.3)	17 (45.9)

At the beginning of the intervention, participants had, on average, worked in a facilitating function for 2.8 years (SD = 1.6, min = 1, max = 6), and as facilitators in their current organization for 2.4 years (SD = 1.4, min = 1, max = 6). Furthermore, the participants were, on average, motivated to learn what was going to be presented in the course (M = 94.1, SD = 9.2, min = 63, max = 100) and were prepared to make a great effort to develop in their facilitator role (M = 88.8, SD = 13.6, min = 44, max = 100). On average, the implementation climate received a score of 57.2 (SD = 19.6, min = 17, max = 94.8). The mean agreement rating with the item measuring collegial support was 57.0 (SD = 23.3, min = 0, max = 100). Pre-intervention mean ratings for each cohort for the variables learning motivation, training motivation, implementation climate, and collegial support are presented in Table 3.

**Table 3:** Pre-intervention mean ratings for learning motivation, training motivation, implementation climate, and collegial support.

Variable	Cohort			Total
	1 (*n* = 10)	2 (*n* = 12)	3 (*n* = 15)	
	Mean (SD)	Mean (SD)	Mean (SD)	Mean (SD)
Learning motivation (single item)	95.5 (5.9)	97.3 (8.3)	89.6 (13.5)	94.1 (9.2)
Training motivation (single item)	93.5 (8.7)	92.5 (16.5)	80.3 (15.8)	88.8 (13.6)
Implementation climate (scale)	68.4 (18.5)	52.6 (20.2)	53.5 (18.1)	57.2 (19.6)
Collegial support (single item)	68.8 (20.2)	52.1 (21.8)	50.2 (27.9)	57.0 (23.3)

### 3.2 Changes in knowledge and skills

The mean score for self-reported knowledge to facilitate implementation pre-intervention was 48.2 (SD = 24.3) whereas the mean score post-intervention was 76.2 (SD = 13.9). There was a statistically significant increase in knowledge to facilitate implementation pre-intervention compared to post-intervention [t(31) = −7.07, *p* < .001]. In the case of skills in facilitation, the mean score pre-intervention was 58.2 (SD = 21.6) and post-intervention 72.6 (SD = 12.5). There was a statistically significant increase in skills in facilitation pre-intervention compared to post-intervention [*t*(31) = −3.82, *p* = .000].

### 3.3 Changes in self-efficacy

**Figure 1:**
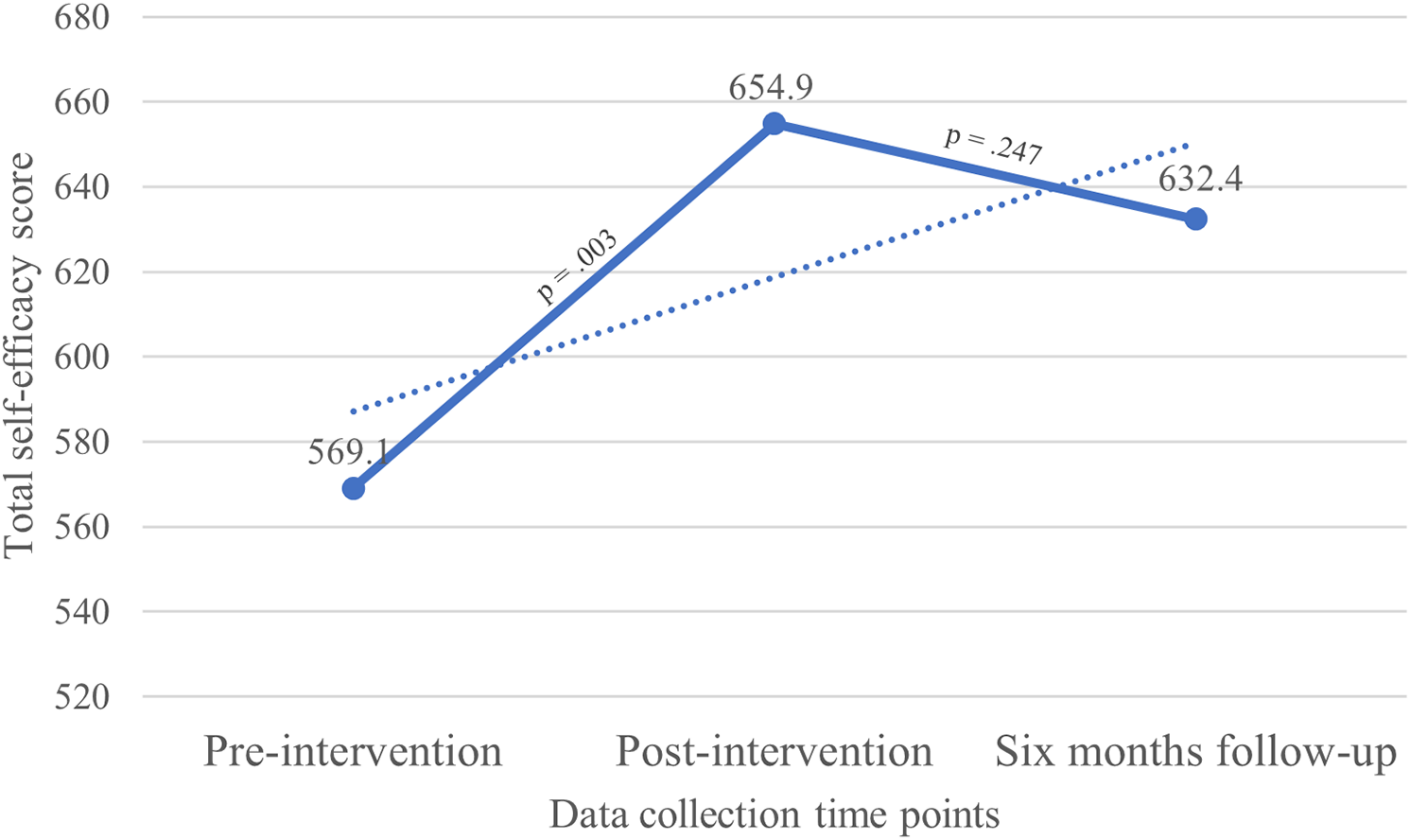
Trends in self-efficacy.

Pre-intervention, the mean rating of participants' self-efficacy was 569.1 (SD = 175.3), post-intervention the mean rating was 655.9 (SD = 146.5), and at six months follow-up the mean rating was 632.4 (SD = 157.4). There was a statistically significant increase in self-efficacy between pre-intervention and the post-intervention measurement (*p* = .003). A statistically nonsignificant decline in mean self-efficacy was observed at the six months follow-up (*p* = .247), however, this decline was not below pre-intervention levels (see Figure 1).

### 3.4 Normalization of the systematic implementation model

The mean ratings of the three general items were: participants' familiarity with the systematic implementation model 6.1 (SD = 2.2); the extent to which the systematic implementation model felt as a natural part of participants' work routine 5.0 (SD = 2.5) and the extent to which the model could become a natural part of the participants' work routine 7.3 (SD = 1.8).

The mean values for the mechanisms coherence, cognitive participation, collective action, and reflexive monitoring were 2.2 (SD = .6), 2.2 (SD = .7), 3.0 (SD = .6) and 2.4 (SD = .7) respectively. Mean values of submechanisms (i.e., items) varied (see Table 4).

**Table 4:** Mean scores and standard deviation of the 16 normalization process theory submechanisms (*on a scale from 1- strongly agree to 5-strongly contradict*) and frequency counts of the responses *not relevant for this role*, *not relevant at the moment,* and *not relevant for this intervention* for each submechanism.

NPT mechanisms and submechanisms^a^	Frequency *n*/*N*	Mean	Standard deviation	Not relevant for this role *n*	Not relevant at the moment *n*	Not relevant for this intervention *n*
**Coherence**		**2**.**2**	**0**.**6**			
Differentiation	17/17	2.2	0.5	0	0	0
Communal specification	12/17	2.7	0.8	0	3	2
Individual specification	16/17	2.1	0.4	0	0	0
Internalization	17/17	1.8	0.6	0	0	0
**Cognitive participation**		**2**.**2**	**0**.**7**			
Initiation	14/17	3.1	1.0	0	3	0
Legitimation	15/17	2.0	0.7	0	2	0
Enrolment	17/17	1.8	0.5	0	0	0
Activation	17/17	1.8	0.4	0	0	0
**Collective action**		**3**.**0**	**0**.**6**			
Interactional workability	16/17	2.5	0.5	0	0	0
Relational integration 1	16/17	2.6	0.6	0	1	0
Relational integration 2	16/17	2.3	0.5	0	1	0
Skillset workability 1	14/17	2.6	0.6	2	1	0
Skillset workability 2	14/17	3.1	0.8	1	2	0
Contextual integration 1	14/17	3.2	0.7	0	3	0
Contextual integration 2	13/17	2.9	0.8	0	4	0
**Reflexive monitoring**		**2**.**4**	**0**.**7**			
Systematization	12/17	3.0	0.8	0	4	0
Communal appraisal	12/17	3.0	0.7	0	4	0
Individual appraisal	14/17	2.1	0.7	0	3	0
Reconfiguration 1	15/17	2.0	0.7	0	2	0
Reconfiguration 2	15/17	2.1	0.6	0	2	0

The values in bold represent the mean and standard deviation for each of the four core mechanisms of NPT: coherence, cognitive participation, collective action, and reflexive monitoring.

^a^ The submechanisms are described in Supplementary Material: Data collection instruments, pp. 13–14.

### 3.5 Qualitative findings

#### 3.5.1 Coherence

##### 3.5.1.1 Differentiation

When comparing the systematic implementation model with their implementation experience, some participants said that before the training they did not have a clear and systematic implementation routine that they followed. The systematic implementation model provided them with a better understanding of the steps needed to carry out a successful implementation. An increased awareness of the planning and follow-up steps, which previously had been overlooked, was reported. Participants who had used other tools to support quality improvement and implementation (e.g., the Plan-Do-Study-Act or project management models) pointed out that the systematic implementation model stood out through its emphasis on specifying the target behavior in detail and on choosing implementation strategies that matched the needs of the staff facilitators supported to do the particular behaviors.

I think what I found useful was the part about how to choose implementation strategies and how to identify needs…To get that structured list [about knowledge, motivation, and opportunities] and use it when analyzing needs…I had never worked in such a structured way before. (Participant 9)

##### 3.5.1.2 Internalization

Participants found the systematic implementation model valuable to their work. The model was commonly described as clear, easy to follow, user-friendly, pedagogical, useful to formulate the right implementation questions and achieve participatory implementation.

What I think is good with this model is that we start from needs and existing conditions, and that we focus on engaging the staff and changing their behavior. (Participant 3)

In some organizations, the systematic implementation model was tested on different projects to decide whether it could be a valuable tool for the organization and if it could be adopted as a formal tool for implementation at the organization level. A challenge experienced by participants was to understand how the model could be used in more complex implementation projects involving many target behaviors and stakeholders positioned at different levels in the organization.

##### 3.5.1.3 Individual specification

Although many participants reported that they felt it was clear what to do when applying the systematic implementation model, some were still testing it in different situations.

I first thought that we would start doing things right away, but it is not like that [according to the model], it is very important to work systematically and have patience, I hadn't initially understood how important this is. (Participant 5)

Two different perspectives were found. According to one perspective, the model should be followed systematically, whereas according to the other, the model would be used as “a source of inspiration” and a degree of flexibility would be maintained when applying it, as not all the steps were considered equally important during implementation.

##### 3.5.1.4 Communal specification

The facilitators who had presented the systematic implementation model to the staff they supported reported different reactions from staff. In some organizations it was easy to achieve a shared understanding of the rationale behind the systematic implementation model and identify the benefits of using it, whereas in other organizations some facilitators were still in the stage of explaining the model's usefulness to the supported staff, with the purpose of creating a common understanding.

We have yet to come so far in the implementation process; I am still trying to motivate [to the others] why we should use the [systematic implementation] model. (Participant 1)

#### 3.5.2 Cognitive participation

##### 3.5.2.1 Initiation

The analysis revealed two approaches employed by facilitators in introducing the systematic implementation model within their respective organizations. One approach was to explicitly present the model to managers or to the staff facilitators supported, to enhance comprehension. Another strategy to increase understanding and engagement was to emphasize the commonalities between the systematic implementation model and another model already used in the organization. The facilitators who had difficulties driving forward the use of the model in their organization, especially at the managerial level, suggested that a workshop leader could support this effort.

Another approach was to communicate knowledge about the model implicitly, for example, by using it to create implementation plans, which they then presented to the staff they supported, by inviting staff to participate in planning days, where they were guided through a discussion about aspects of implementation, or by signaling ahead of time the upcoming implementation steps and that these were important.

I have not talked about the whole model [to the supported staff], but I have said that when implementing something new, we need to focus on behavior change. (Participant 13)

##### 3.5.2.2 Enrolment

The facilitators observed varying levels of staff enrolment in the work with the systematic implementation model. Enrolment, in this context, refers to the degree of staff engagement and commitment to the model. In organizations where enrolment was high, staff demonstrated a strong commitment to the model, showing enthusiasm and readiness to integrate it into their daily work. In organizations where staff enrolment was low, facilitators quoted time constraints as a significant barrier to convening staff for collaborative implementation planning sessions.

Bringing the group to the same starting point is the first step and we have never taken the time and progressed at this pace. Just bringing together my colleagues, who are also supposed to lead this [implementation], has not been easy; we have always been expected to do things right away, and they should have been done yesterday and we should have results tomorrow. (Participant 2)

##### 3.5.2.3 Activation

Some interview participants expressed their commitment to using the systematic implementation model in other projects, but they emphasized the need to find time to reflect on how to do this in the future.

I hope that I will have the time to reflect on this [the use of the systematic implementation model in future projects], but right now, I'm not working with an [implementation] task…a lot happens all the time and, at the moment, I work with patients. So, right now, I don't have the time to sit and read and think. (Participant 12)

#### 3.5.3 Collective action

##### 3.5.3.1 Interactional workability

Echoing the findings reported in *Individual specification*, the systematic implementation model was applied to implementation projects ranging from small to large, where some participants applied it mainly to smaller implementation projects and others only to larger projects. For example, facilitators used the model to ask the group relevant questions that led to project replanning, to systematize the implementation process, and to help staff reframe their thinking in terms of behaviors.

The systematic implementation model was seldom used in its entirety. Common reasons for not following the model closely were a lack of time to go through every step and the increased complexity of the implementation plan if the model was to be used for every stakeholder. Needs analysis, specification of target behavior, and follow-up were the most used steps.

What I have specifically used is this about how to clarify target behaviors…which behavior do we really want to look at…this should be made clear. Also following up…yes, that we should follow up the target behaviors. Because this is, actually, the most difficult…to follow up constructively. (Participant 10)

##### 3.5.3.2 Skillset workability

There were descriptions of staff in organizations, both directly responsible for the implementation of the project but also from support units (communications), who had either begun or were about to participate in the training provided by CES. Most facilitators were positive towards contributing to building the general implementation capacity of their organizations by equipping staff and managers with the knowledge and skills to use the systematic implementation model. However, some participants felt the need to reflect and plan how to proceed with the task, while emphasizing the importance of staff members attending the training rather than solely relying on facilitators for their training needs.

I don't know if I would be the best to do it [train other staff in implementation]. The training contained so much good information that I would rather they [other staff] participate in the training to get the same aha-experiences and understandings. [The information] is quite easy to understand, but it has to be concretized in the same way it was presented in the training so I would rather that they take the course instead. (Participant 2)

Facilitating implementation alone was seen as challenging, and several facilitators wished they could work alongside others who shared their role and had undergone the same implementation training, ensuring a shared understanding of the process.

##### 3.5.3.3 Contextual integration

Participants' work with the systematic implementation model was carried out during the Covid-19 pandemic. Most facilitators operated within high-pressure environments, with significant staff turnover or understaffing. Some contexts were characterized by lengthy decision processes involving decision-makers at different organizational levels, work cultures where improvement was not valued, or low prioritization of implementation work.

The contextual integration of the systematic implementation model was highly influenced by managerial support. Managers backed the participants' work with the model to different extents. Support was provided through different means, from giving an assignment to work with implementation, to more direct involvement from managers, such as clear communication around the importance of implementation and its prioritization in the organization, and by bringing it for discussions in different forums, such as staff meetings.

It [the systematic implementation model] has been received positively, and I have noticed that, for example, in the manager group, a maturation phase has begun regarding this way of thinking; I am no longer the only one that goes around and says that these things are important and nobody else really understands. So, I think that it [the systematic implementation model] has contributed to a maturation of the organization [around the implementation process]. (Participant 7)

However, not all facilitators shared this experience. There were also descriptions of absence of support, attributed to the managers' low general knowledge of implementation, which, for example, translated into a lack of strategic planning, favoring short-term projects that were not followed up, and not focusing on behavior change. One strategy that was often suggested to overcome this problem was to provide staff and management with some basic training in implementation, which would increase their understanding of the implementation process and the conditions in which it is successful.

#### 3.5.4 Reflexive monitoring

##### 3.5.4.1 Systematization

Participants recognized the significance of conducting follow-ups and assessments of the application of the systematic implementation model. Nonetheless, they highlighted that these are hindered by persistent conflicting demands.

According to the model, one should continue to follow up and evaluate and give feedback, so that it continues improving [and it becomes part of routine], but it is hard when there are more and more things to do all the time. (Participant 9)

##### 3.5.4.2 Individual appraisal

A few participants mentioned they had appraised the model. Often the appraisal was done individually by the facilitators, who generally reported positive evaluations of its usefulness.

I think that has been really good and a great complement to PDSA and all that I worked with earlier. (Participant 13)

##### 3.5.4.2 Communal appraisal

Participants working in organizations where the model was being tested for organizational fit reported that project staff considered it a valuable tool after testing it in a pilot project.

As I understand it, those in the pilot project feel that it works very well, and they are very interested in taking the training. (Participant 4)

##### 3.5.4.3 Reconfiguration

Reconfiguring the systematic implementation model after a formal appraisal was rare. Adaptations to the model were made ongoingly, to fit the needs of current implementations, rather than after a formal evaluation at the end of an implementation cycle (see also *interactional workability*).

I can say that for us, it hasn't been crucial to follow all the steps. Sometimes, we moved forward by understanding that we need to concentrate most on this first step. Then, we implement, and someone else takes over to ensure that the wheel, equivalent to the PDSA wheel, keeps turning. Facilitators can then be there to follow up. So, I think something that another facilitator who participated in the training with me and I learned was that we can orient ourselves…not freely, but we can emphasize different steps [of the systematic implementation model] depending on what we need to achieve. (Participant 1)

## 4 Discussion

This study evaluated the BIC-F intervention, which aimed to train individuals who hold a facilitating role in organizations. The results, which we will discuss in more detail in subsequent paragraphs, indicate: (1) Statistically significant increases in participants’ perceptions of their knowledge and skills to facilitate implementation and their self-efficacy as a facilitator post-intervention, compared to pre-intervention; and (2) An early stage in the process of normalization of the systematic implementation model in the participants' work routines, with strong coherence but relatively low activation of other NPT mechanisms, and at the organizational level, where many facilitators were still working towards achieving coherence and cognitive participation among relevant stakeholders.

Earlier research has shown that self-efficacy is a good indicator of behavioral modification, and increasing participants’ self-efficacy regarding one or a set of tasks may improve the odds that they perform the task(s) even after the training (36). The importance of self-efficacy in training transfer is also suggested by previous research, which has studied it as an outcome of capacity-building interventions (44). In our study, the statistically significant increase in participants' self-efficacy post-intervention could result from the strong emphasis placed on practice throughout the intervention. Specifically, the participants practiced creating implementation plans for several cases and took part in role-playing exercises. These activities could have served as sources of self-efficacy in the form of *mastery experience* (also called performance accomplishments), which seems to be the most effective way of developing self-efficacy (45). Another source of self-efficacy that may have been activated during the training was *vicarious experience*, which entails learning from other participants during group discussions and presentations and by observing workshop leaders apply the knowledge during workshops (45).

Self-efficacy is strengthened by repeated success, achieved through practice (46, 47). The decrease in self-efficacy scores between the first post-intervention measurement and the second post-intervention measurement could be explained, in part, by limited opportunities to apply the knowledge and skills participants acquired in the training after the intervention, as reported in some interviews. Factors external to the organization (e.g., the Covid-19 pandemic) as well as internal to the organization (e.g., lack of prioritization of implementation work) were mentioned as limiting conditions. Previous research has suggested that facilitators can practice their role better if organizations prioritize training and implementation work (48). It is, thus, likely that the professional development of facilitators who undergo even high-quality training is hindered without regular practice, which can only happen under auspicious organizational conditions. Increased contact between the workshop leaders and the facilitators could help the latter maintain momentum after the intervention. Furthermore, workshop leaders and facilitators could find strategies targeted at management to increase the prioritization of implementation work in organizations.

The results of this study indicate that the participants were in the early phases of normalizing the systematic implementation model in their work routines. Six months after the intervention, participants, on average, were not fully familiar with the model, and for many, it did not feel like a natural part of their work routines. However, it was stated that the model could become part of their work routines. There was a general understanding of the steps and value of the model for their implementation work, which was suggested by low S-NoMAD scores on the differentiation, individual specification, and internalization items (lower scores suggest higher contribution to normalization), and by positive interview accounts referring to these submechanisms of *coherence*. This aligns with previous research showing that an implementation process starts with achieving coherence (49). In light of qualitative data describing that only a few facilitators used the implementation model systematically, the medium rating on *interactional workability* suggests that the normalization process was in its early stages for many participants. A too short follow-up period (one year) could partly explain this finding, given that a similar study noted that change in behavior and applied knowledge in their sample typically occurred 18–24 months after the intervention (50). The qualitative data give further insight into how participants applied the systematic implementation model. Not all participants utilized all the steps in the model, which is consistent with findings from a prior study that evaluated an intervention teaching the model (51). In our study, a detailed application of the model was perceived by some participants as making the planning process too difficult. This might clarify why a few facilitators were still grappling with how to apply the model to more complex implementation projects. Using the model flexibly or as inspiration was also suggested in a few interviews. This type of use could be understood according to recent theoretical perspectives, which underscore the significance of considering mental models when describing how individuals approach implementation (36). Mental models can determine how information is understood and what changes individuals will consider, and they are shaped over time by continuous learning, experiences, and exposure to different contexts (52), which participants in our study reported. This lends support to the idea that facilitation involves flexibility in applying and blending different approaches (6) and that the “implementation of implementation science knowledge” (54), like any other type of scientific knowledge, will not be linear and straightforward, but rather the subject of individual interpretation and contextual adaptation (55).

Apart from their role in supporting the implementation of EBIs, facilitators can also contribute to building an organization's implementation capacity (6, 16). Intervention participants were encouraged to share the learnings from the training and engage other staff in implementation planning. Medium scores on S-NoMAD items assessing the coherence and engagement of other staff in using the systematic implementation model (e.g., communal specification, initiation) and perceived organizational support for the use of the model (e.g., contextual integration) suggest that the process of its normalization was not very advanced in most organizations. In interviews, there were examples indicating that participants did not mention the model at all, mentioned it briefly, or just created implementation plans based on the model, which they then shared with the group they supported. The limited dissemination of knowledge and engagement of other staff in applying the systematic implementation model might be, to some extent, clarified by the facilitators' need to first become more comfortable with using the model in their routines. This finding is in line with a previous study where *learning over time* was a mechanism that led to an increased sense of confidence and an improved ability to enact the facilitation role (20), and could also account for why some participants responded with *Not relevant at the moment* on S-NoMAD items related to collaborative work with other staff members. Additional factors that could have hindered the use of the model in some organizations, as indicated by pre-intervention data and qualitative results, were implementation climate and collegial support.

### 4.1 Curriculum development

The feedback from the evaluation regarding the need to understand how to apply the systematic implementation model to more complex implementation cases resulted in the addition of another module to the training. The module consisted of a series of lectures and exercises on complex implementation where participants gained a deeper understanding of how to facilitate the implementation of interventions with several components at different levels of an organization.

The evaluation highlights that the BIC-F intervention has had a positive impact on participants' knowledge, skills, and self-efficacy, most likely due to its focus on practical application of implementation and facilitation knowledge throughout the workshops. Nevertheless, future iterations of the BIC-F intervention could emphasize the role facilitators can play in building the implementation capacity of the organizations, along with their role in facilitating the implementation of EBIs. Furthermore, additional components that explain how implementation knowledge and tools can intentionally be transferred between the different levels of the organization (12) could provide a starting point for facilitators to achieve a common understanding about implementation with staff and managers and a greater collective engagement and support necessary to develop the organizations' implementation capacity. Finally, the findings suggest that workshop leaders could positively influence the implementation climate in some organizations by engaging with upper management and advocating for the adoption of a more systematic approach to implementation.

## 5 Strengths and limitations

A strength of this study was its mixed methods sequential explanatory approach, where qualitative data were used to provide possible explanations of the quantitative results. The longitudinal design involving several data collection timepoints is also a strength, as it allowed us to understand better how participants used the knowledge and skills they acquired in the training, what challenges they encountered even after the intervention ended, and the type of support they could benefit from. Furthermore, the diversity of participants in terms of their roles, responsibilities, and organizations indicates that this training might be relevant for a wide range of professionals activating within healthcare and other public services and strengthens the external validity of the study.

The study results should be interpreted considering a series of limitations. An important limitation is the lack of a control group, which impeded us from estimating the effect size of the intervention. Another limitation was the attrition of study participants over time. This could have been because of self-selection bias, leading to not capturing other outcomes and perspectives that might have provided a more detailed understanding of the participants' experiences of the intervention and its results. Furthermore, the extraordinary circumstances in which the intervention was delivered (i.e., the Covid-19 pandemic) and the one-year follow-up period are also considerable limitations which could have impacted the results. However, given that this was a pilot study, a one-year follow-up period was considered sufficient to observe some meaningful changes and to guide a longer evaluation. Another weakness is that the quantitative data were collected using self-reported measures, which could have led to an under- or overestimation of the measured constructs. Furthermore, responses in interviews could have been affected by social desirability bias, but this risk was mitigated by not involving the interviewer in the delivery of the intervention.

Using NPT to frame the longitudinal evaluation and as an aid in interpreting the results is a strength of this study, as the theory has been previously found to provide a robust explanation of how interventions are embedded in practice. However, the deductive approach we used to analyze the qualitative data highlighted overlaps between NPT constructs, which was also observed by other studies (49). This occasionally made coding meaning units with a single NPT construct challenging. For example, individual specification and internalization necessarily required applying the systematic implementation model (i.e., interactional workability). In some cases, interactional workability was also concomitant to reconfiguring the model. Furthermore, NPT is a relational theory, which was relevant for interpreting the perspectives of the facilitators, who are professionals who often initiate and create support for implementation through relational work. Nonetheless, collecting data only from facilitators limited our understanding of the normalization process of the model at the level of the organization, as we tried to make sense of collective processes such as communal specification and enrolment based only on the participants' points of view.

## 6 Conclusion

The intervention seemed to improve participants' knowledge, skills, and self-efficacy, and it started the process of normalizing the systematic implementation model in the participants' work routines. Nevertheless, in most cases, the normalization process was in the early stages and was often not approached in an intentional and systematic way because personal or organizational factors hindered transfer of training. To ensure improved transfer of training and higher normalization of the methods they teach, the BIC-F and other interventions need to provide long-term support to facilitators, and include components designed to tackle contextual obstacles and actively build implementation capacity at the organizational level.

## Data Availability

The raw data supporting the conclusions of this article will be made available by the authors, without undue reservation.
