# Supplementary material for: A longitudinal mixed methods evaluation of a facilitation training intervention to build implementation capacity
Source: Front Health Serv. 2024 Sep 13;4:1408801. doi: 10.3389/frhs.2024.1408801 (PMC11427355; doi:10.3389/frhs.2024.1408801)
Supplement: Supplementary file 2 [file Table2.docx]

# Interview guide

## Questions about experiences of working as a facilitator before and after the training

1. **Can you tell me about your role in your organization?** Does the role involve a facilitating function?
2. **What were your experiences of working in a facilitating function before participating in the training?** Which knowledge and skills did do you usually need in your role to support implementation? How did you experience working in a facilitating function before participating in the training? What did you find challenging? How long have you had this role?
3. **Why did you apply to the training?**
4. **How did you experience the role as a facilitator after the training?**

**The training presented different topics, such as an introduction to implementation, including how a systematic implementation model is used, how to communicate, and how to manage resistance to change. Have you used any of the knowledge from the training?** What have you specifically used? In which contexts? **Has the training impacted your ability to act in a facilitating function?** In which way? **If knowledge from the training hasn’t been used:** Why not? Which information did you miss?

## Questions about the normalization of the implementation model

1. **What do you think about the systematic implementation model?**
2. **How has it been to use the systematic implementation model to support implementation in your organization?** Follow-up questions if the systematic implementation model hasn’t been used: Why not? Have you used other models? Have there been organisational factors that hinder the use of the model?
3. **How is working with the systematic implementation model different from how you have worked with implementation before the training?**
4. **How has using the systematic implementation model impacted your work routine?** Which factors have facilitated the use of the model in your work routine? Which factors have hindered the use of the model in your work routine?
5. **Have you shared information about the systematic implementation model to the individuals that you support in the process of implementation in your organization?** How?
6. **How has the systematic implementation model been received by the individuals that you support in the process of implementation?** Have they understood the purpose of using the systematic implementation model? If the systematic implementation model hasn’t been shared with the group: Why not? How do you see your role to develop systematic implementation routines in the organizations where you work as a facilitator?
7. **How involved in working with implementation are other people in your organization?** What do you think about having a role in training others in the organization to use the systematic implementation model? Are individuals in the groups you support interested in receiving such training?
8. **Which resources are needed to support the use of the systematic implementation model in the organization where you work as a facilitator?**
9. **What does the managers think about the use of the systematic implementation model for implementation in your organization?** Which support have you received from your managers?
10. **Do you need additional information or support to use or continue using the systematic implementation model?**
11. **How do the staff you support perceive continuing to use the systematic implementation model for upcoming implementations?**
12. **Have you adapted the systematic implementation model in any way to meet your needs better? In what way?**
13. **Is there anything you would like to add that can increase my understanding about how you have experienced your role and your work as a facilitator after participating in the training Building implementation capacity for facilitation?**

# Pre-intervention questionnaire

## My attitude towards the training *Building implementation capacity for facilitation*

*Rate your attitude regarding the following statements:*

1. I have high expectations that the course can increase:

1a. my knowledge to support the implementation of new work routines.

Completely disagree Completely agree

1b. my skills to support the implementation of new work routines.

Completely disagree Completely agree

1. I am convinced that I will be able to use what I learn during the training in my organization.

Completely disagree Completely agree

1. I look forward to participating in the training.

Completely disagree Completely agree

1. I am ready to accept the changes that will take place in my organisation as a result of what I learn during the course.

Completely disagree Completely agree

1. I am motivated to learn what is presented in the course.

Completely disagree Completely agree

1. I am prepared to make a big effort to develop myself in the role of facilitator.

Completely disagree Completely agree

1. I am convinced that, currently, in my role as facilitator:

7a. I can always manage to solve difficult problems if I try hard enough.

Completely disagree Completely agree

7b. If someone opposes me, I can find the means and ways to get what I want.

Completely disagree Completely agree

7c. It is easy for me to stick to my aims and accomplish my goals.

Completely disagree Completely agree

7d. I am confident that I could deal efficiently with unexpected events.

Completely disagree Completely agree

7e. thanks to my resourcefulness, I know how to handle unforeseen situations.

Completely disagree Completely agree

7f. I can solve most problems if I invest the necessary effort.

Completely disagree Completely agree

7g. I can remain calm when facing difficulties because I can rely on my coping abilities.

Completely disagree Completely agree

7h. When I am confronted with a problem, I can usually find several solutions.

Completely disagree Completely agree

7i. If I am in trouble, I can usually think of a solution.

Completely disagree Completely agree

7j. I can usually handle whatever comes my way.

Completely disagree Completely agree

## Perceptions of my competence

*In this questionnaire the word “target group” refers to staff, managers, and other key individuals in the organisation where you work as a facilitator.*

1. I have enough knowledge of facilitation to be able to support target groups to:

8a. plan the implementation of new work routines.

Completely disagree Completely agree

8b. carry out appropriate strategies to support implementation of new work routines.

Completely disagree Completely agree

8c. follow up the implementation of new work routines.

Completely disagree Completely agree

8d. adapt the implementation of new work routines to the organization where I work.

Completely disagree Completely agree

8e. coordinate the implementation of new work routines.

Completely disagree Completely agree

1. I have enough skills to:

9a. motivate the target group to implement work routines.

Completely disagree Completely agree

9b. communicate the implementation of a new work routine clearly.

Completely disagree Completely agree

9c. express understanding for the problems the target group experiences.

Completely disagree Completely agree

9d. manage conflicts.

Completely disagree Completely agree

9e. give constructive feedback in relation to the current implementation.

Completely disagree Completely agree

1. The target group that I currently support:

10a. is expected to implement new work routines.

Completely disagree Completely agree

10b. receives the support they need to implement new work routines.

Completely disagree Completely agree

10c. receives positive feedback when they implement new work routines.

Completely disagree Completely agree

10d. believe it is important to change work routines when needed to achieve the best possible service quality.

Completely disagree Completely agree

10e. prioritize changing work routines when needed to achieve the best possible service quality.

Completely disagree Completely agree

1. I believe that the target group will support me to apply the competencies I learn during the course.

Completely disagree Completely agree

1. Number of years I have worked in a facilitating function: __________

# Post-intervention questionnaire

## My attitude towards the training *Building implementation capacity for facilitation*

*Rate your attitude regarding the following statements:*

1. I have high expectations that the training can increase:

1a. my knowledge to support implementation of new work routines.

Completely disagree Completely agree

1b. my skills to support implementation of new work routines.

Completely disagree Completely agree

1. I am motivated to apply what I learned in the course in my work as a facilitator.

Completely disagree Completely agree

1. After participating in the training, I am convinced that, in my role as facilitator:

3a. I can always manage to solve difficult problems if I try hard enough.

Completely disagree Completely agree

3b. If someone opposes me, I can find the means and ways to get what I want.

Completely disagree Completely agree

3c. It is easy for me to stick to my aims and accomplish my goals.

Completely disagree Completely agree

3d. I am confident that I could deal efficiently with unexpected events.

Completely disagree Completely agree

3e. thanks to my resourcefulness, I know how to handle unforeseen situations.

Completely disagree Completely agree

3f. I can solve most problems if I invest the necessary effort.

Completely disagree Completely agree

3g. I can remain calm when facing difficulties because I can rely on my coping abilities.

Completely disagree Completely agree

3h. When I am confronted with a problem, I can usually find several solutions.

Completely disagree Completely agree

3i. If I am in trouble, I can usually think of a solution.

Completely disagree Completely agree

3j. I can usually handle whatever comes my way.

Completely disagree Completely agree

## Perceptions of my competence

*In this questionnaire the word “target group” refers to staff, managers, and other key individuals in the organisation where you work as a facilitator.*

1. I have enough knowledge of facilitation to be able to support target groups to:

4a. plan the implementation of new work routines.

Completely disagree Completely agree

4b. carry out appropriate strategies to support implementation of new work routines.

Completely disagree Completely agree

4c. follow up the implementation of new work routines.

Completely disagree Completely agree

4d. adapt the implementation of new work routines to the organization where I work.

Completely disagree Completely agree

4e. coordinate the implementation of new work routines.

Completely disagree Completely agree

1. I have enough skills to:

5a. motivate the target group to implement new work routines.

Completely disagree Completely agree

5b. communicate the implementation of a new work routine clearly.

Completely disagree Completely agree

5c. express understanding for the problems the target group experiences when implementing new work routines.

Completely disagree Completely agree

5d. manage conflicts.

Completely disagree Completely agree

5e. give constructive feedback in relation to the current work routine implementation.

Completely disagree Completely agree

# Follow-up questionnaire (6 months)

## Questions about yourself

|  | Less than a year | 1-2 years | 3-5 years | 6-10 years | 11-15 years | More than 15 years |
| --- | --- | --- | --- | --- | --- | --- |
| How long have you worked in a facilitating function? |  |  |  |  |  |  |
| How long have you worked in a facilitating function in your organization? |  |  |  |  |  |  |

## Rate your attitude regarding the following statements

**Currently, in my role as a facilitator, I am convinced that:**

1. I can always manage to solve difficult problems if I try hard enough.

Completely disagree Completely agree

1. If someone opposes me, I can find the means and ways to get what I want.

Completely disagree Completely agree

1. It is easy for me to stick to my aims and accomplish my goals.

Completely disagree Completely agree

1. I am confident that I could deal efficiently with unexpected events.

Completely disagree Completely agree

1. Thanks to my resourcefulness, I know how to handle unforeseen situations.

Completely disagree Completely agree

1. I can solve most problems if I invest the necessary effort.

Completely disagree Completely agree

1. I can remain calm when facing difficulties because I can rely on my coping abilities.

Completely disagree Completely agree

1. When I am confronted with a problem, I can usually find several solutions.

Completely disagree Completely agree

1. If I am in trouble, I can usually think of a solution.

Completely disagree Completely agree

1. I can usually handle whatever comes my way.

Completely disagree Completely agree

## A. General questions about the use of the implementation model

*The following questions refer to the systematic implementation model, shown below:*


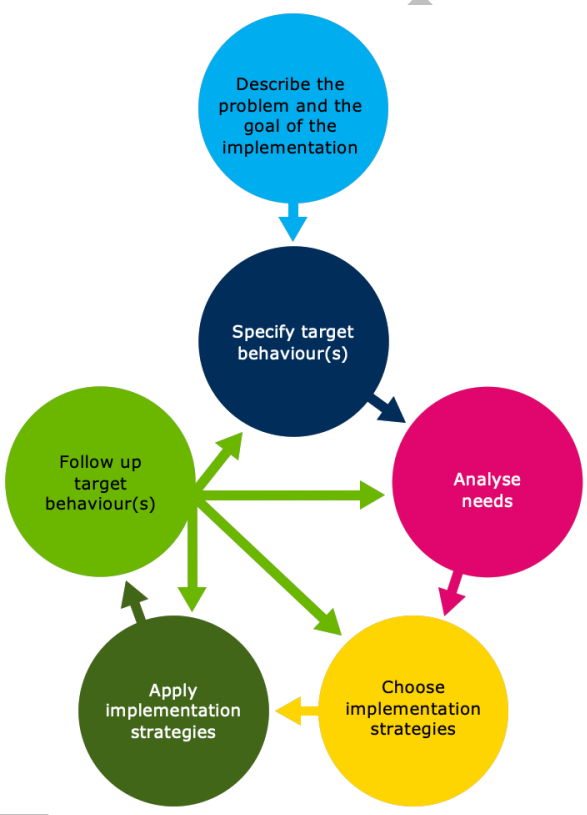


A1. When you use the systematic implementation model, how familiar does it feel to you?

Not at all familiar Very familiar

A2. Do you feel that the systematic implementation model is a natural part of your work routine?

Not at all Completely

A3. Do you believe that the systematic implementation model can become a natural part of your work routine?

Not at all Completely

## B. Detailed questions about the use of the systematic implementation model in your role as a facilitator

**For each affirmation, choose the answer that matches your experience by using the alternatives 1 (Completely agree) to 5 (Completely contradict). If the affirmation is not relevant for you, choose an answer from the last three alternatives.**

|  | 1  Completely agree | 2  Agree | 3  Neither agree nor contradict | 4  Contradict | 5  Completely contradict | 6  Not relevant for this role | 7  Not relevant at the moment | 8  Not relevant for the intervention |
| --- | --- | --- | --- | --- | --- | --- | --- | --- |
| B1. I can see how the use of the systematic implementation model differs from usual ways of working.  (Differentiation) |  |  |  |  |  |  |  |  |
| B2. Staff in this organisation have a shared understanding of the purpose of this systematic implementation model.  (Communal specification) |  |  |  |  |  |  |  |  |
| B3. I understand how the systematic implementation model affects the nature of my own work.  (Individual specification) |  |  |  |  |  |  |  |  |
| B4. I can see the potential value of the systematic implementation model for my work. (Internalization) |  |  |  |  |  |  |  |  |
| B5. There are key people who drive the systematic implementation model forward and get others involved.  (Initiation) |  |  |  |  |  |  |  |  |
| B6. I believe that participating in the systematic implementation model is a legitimate part of my role.  (Legitimation) |  |  |  |  |  |  |  |  |
| B7. I’m open to working with colleagues in new ways to use the systematic implementation model.  (Enrolment) |  |  |  |  |  |  |  |  |
| B8. I will continue to support the systematic implementation model.  (Activation) |  |  |  |  |  |  |  |  |
| B9. Interactional workability I can easily integrate the systematic implementation model into my existing work.  (Interactional workability) |  |  |  |  |  |  |  |  |
| B10. The systematic implementation model disrupts working relationships.  (Relational integration 1) |  |  |  |  |  |  |  |  |
| B11. I have confidence in other people’s ability to use the systematic implementation model.  (Relational integration 2) |  |  |  |  |  |  |  |  |
| B12. Work is assigned to those with skills appropriate to the systematic implementation model.  (Skillset workability 1) |  |  |  |  |  |  |  |  |
| B13. Sufficient training is provided to enable staff to use the systematic implementation model.  (Skillset workability 2) |  |  |  |  |  |  |  |  |
| B14. Sufficient resources are available to support the systematic implementation model.  (Contextual integration 1) |  |  |  |  |  |  |  |  |
| B15. Management adequately support the systematic implementation model.  (Contextual integration 2) |  |  |  |  |  |  |  |  |
| B16. I am aware of reports about the effects of the systematic implementation model.  (Systematization) |  |  |  |  |  |  |  |  |
| B17. The staff agree that the systematic implementation model is worthwhile.  (Communal appraisal) |  |  |  |  |  |  |  |  |
| B18. I value the effects the systematic implementation model has had on my work.  (Individual appraisal) |  |  |  |  |  |  |  |  |
| B19. Feedback about the systematic implementation model can be used to improve it in the future.  (Reconfiguration 1) |  |  |  |  |  |  |  |  |
| B20. I can modify how I work with the systematic implementation model.  (Reconfiguration 2) |  |  |  |  |  |  |  |  |
